# Thiamine metabolism genes in diatoms are not regulated by thiamine despite the presence of predicted riboswitches

**DOI:** 10.1111/nph.18296

**Published:** 2022-07-01

**Authors:** Marcel Llavero‐Pasquina, Katrin Geisler, Andre Holzer, Payam Mehrshahi, Gonzalo I. Mendoza‐Ochoa, Shelby A. Newsad, Matthew P. Davey, Alison G. Smith

**Affiliations:** ^1^ Department of Plant Sciences University of Cambridge Downing Street Cambridge CB2 3EA UK; ^2^ Scottish Association of Marine Sciences Oban PA37 1QA UK

**Keywords:** aptamer prediction, CRISPR/Cas9, diatoms, *Phaeodactylum tricornutum*, thiamine biosynthesis, thiamine uptake, TPP riboswitch

## Abstract

- Thiamine pyrophosphate (TPP), an essential co‐factor for all species, is biosynthesised through a metabolically expensive pathway regulated by TPP riboswitches in bacteria, fungi, plants and green algae. Diatoms are microalgae responsible for *c.* 20% of global primary production. They have been predicted to contain TPP aptamers in the 3′UTR of some thiamine metabolism‐related genes, but little information is known about their function and regulation.
- We used bioinformatics, antimetabolite growth assays, RT‐qPCR, targeted mutagenesis and reporter constructs to test whether the predicted TPP riboswitches respond to thiamine supplementation in diatoms. Gene editing was used to investigate the functions of the genes with associated TPP riboswitches in *Phaeodactylum tricornutum*.
- We found that thiamine‐related genes with putative TPP aptamers are not responsive to supplementation with thiamine or its precursor 4‐amino‐5‐hydroxymethyl‐2‐methylpyrimidine (HMP), and targeted mutation of the TPP aptamer in the *THIC* gene encoding HMP‐P synthase does not deregulate thiamine biosynthesis in *P. tricornutum*. Through genome editing we established that *PtTHIC* is essential for thiamine biosynthesis and another gene, *PtSSSP*, is necessary for thiamine uptake.
- Our results highlight the importance of experimentally testing bioinformatic aptamer predictions and provide new insights into the thiamine metabolism shaping the structure of marine microbial communities with global biogeochemical importance.

## Introduction

Thiamine pyrophosphate (TPP), the biologically active form of thiamine (vitamin B_1_), acts as a co‐factor for key enzymes such as pyruvate dehydrogenase, transketolase and pyruvate decarboxylase, and is an essential micronutrient for virtually all organisms (Hanson *et al*., 2018). The widespread use of thiamine across all kingdoms of life suggests a long evolutionary history and supports the hypothesis that B vitamins are remnants of the first organic catalysts in the RNA world (White, 1976). Thiamine pyrophosphate is biosynthesised *de novo* via the condensation of two intermediates: 4‐amino‐5‐hydroxymethyl‐2‐methylpyrimidine pyrophosphate (HMP‐PP) and 4‐methyl‐5‐(2‐phosphooxyethyl)thiazole (HET‐P), but the production of these precursors follows alternative routes in different kingdoms (Webb *et al*., 2007). In prokaryotes, plants and green algae, the pyrimidine moiety is produced from 5‐aminoimidazole ribotide (AIR) by HMP‐P synthase (THIC), whereas in fungi it is produced from pyridoxal‐5‐phosphate and histidine by THI5/NMT1 (Coquille *et al*., 2012). HET‐P is produced in eubacteria via ThiG from iminoglycine, pyruvate, glyceraldehyde‐3‐phosphate and cysteine, and in archaea, fungi, plants and green algae it is produced via THI1/THI4 using NAD^+^, glycine and a sulphur atom from a cysteine residue in the active site (Jurgenson *et al*., 2009). THI4 is therefore a suicide enzyme only capable of a single turnover, which is also true for THI5/NMT1 (Lai *et al*., 2012). Moreover, THIC has a very low turnover rate and is inhibited by the 5′‐deoxyadenosine radical intermediate (Palmer & Downs, 2013), making thiamine biosynthesis a metabolically expensive process (Hanson *et al*., 2018).

Many microbial species, including bacteria and algae, have lost the ability to produce thiamine *de novo*, therefore reducing metabolic costs. But, in return, this renders them dependent on an environmental source of the vitamin or one or more of its precursors (Croft *et al*., 2006). Within the algal lineages, thiamine auxotrophy has evolved multiple times (Helliwell *et al*., 2013) and is widespread in bloom‐forming algae, including the picoeukaryotic prasinophytes, such as *Ostreococcus tauri,* and dinoflagellates. It has been hypothesised that environmental levels of thiamine and its precursors shape the behaviour of algal blooms and determine microbial community structure with significant implications for oceanic ecosystems and global biogeochemical cycles (Bertrand & Allen, 2012; Gutowska *et al*., 2017). Thiamine auxotrophy is less common in diatoms, a major group of marine microalgae responsible for up to 20% of global primary productivity (Field *et al*., 1998; Rousseaux & Gregg, 2014). Interestingly, these organisms are thought to produce HMP‐PP via THIC through a pathway homologous to plants and green algae, but produce HET‐P through the bacterial pathway reliant on ThiG (Bertrand & Allen, 2012).

The high metabolic cost of thiamine biosynthesis might also explain the presence of feedback regulation mechanisms in species with a complete biosynthetic pathway. We have previously demonstrated that, in the presence of exogenous thiamine, the green alga *Chlamydomonas reinhardtii* downregulates the expression of *THIC* and *THI4* via TPP riboswitches, regulatory elements in mRNA that upon direct binding of a ligand, in this case TPP, trigger a change in genetic expression (Croft *et al*., 2007; Moulin *et al*., 2013). Similarly, riboswitches control *THIC* in plants (Wachter *et al*., 2007) and both *THI5/NMT1* and *THIA* (equivalent to *THI1/THI4*) in fungi (Cheah *et al*., 2007). Riboswitches contain two functional units: the aptamer and the expression platform (Roth & Breaker, 2009). The aptamer binds a given ligand with high specificity and often with equilibrium dissociation constants (KDs) in the nanomolar range. Upon binding the substrate, the aptamer undergoes a conformational change that is transduced by the associated expression platform into a change of gene expression. In bacteria, in which riboswitches responsive to a range of metabolite ligands are widespread, the expression platform mechanism can involve masking the ribosome binding site, the start codon or termination elements (Rodionov *et al*., 2002). In eukaryotes, all examples characterised to date are those that respond to TPP, in a mechanism involving alternative splicing (Nguyen *et al*., 2016).

In the past decade, several bioinformatic approaches have been developed to identify putative riboswitches based on sequence information. For instance, Croft *et al*. (2007) analysed sequence conservation between the noncoding regions of the *THIC* gene in the diatoms *Phaeodactylum tricornutum* and *Thalassiosira pseudonana* and identified the presence of a putative TPP riboswitch aptamer in the *THIC* 3′ untranslated region (3′UTR). Later, McRose *et al*. (2014) used the conserved functional motif ‘CUGAGA’ as query against transcripts of thiamine‐related genes in combination with secondary structure predictions and homology searches to identify putative riboswitches in the genomes of a wide variety of eukaryotic supergroups (alveolates, stramenopiles, rhodophytes, rhizaria, chlorophytes, prasinophytes, cryptophytes and haptophytes). In their study, they identified putative riboswitches for *THIC* and for *SSSP*, encoding a predicted thiamine transporter, in diatoms *P. tricornutum*, *T. pseudonana*, *Fragilariopsis cylindrus* and *Pseudonitzschia multiseries*.

In diatoms, both the thiamine biosynthetic pathway and the presence of TPP aptamers have been predicted using bioinformatic methods, but they have not been experimentally studied before. Here, we experimentally test whether the predicted TPP riboswitches found in diatoms *P. tricornutum* and *T. pseudonana* regulated thiamine biosynthesis at the transcript, protein and intracellular thiamine levels using wild‐type and reporter strains. In addition, we use a CRISPR/Cas9 approach to test the predicted function of genes containing TPP riboswitches in *P. tricornutum*.

## Materials and Methods

### Strains and culture conditions

*Phaeodactylum tricornutum* CCAP 1055/1 was grown in f/2 minus silica without vitamins at 18°C and 30 μmol m^−2^ s^−1^ under a 16 h : 8 h, day : night cycle. *Thalassiosira pseudonana 1085/12* was grown in f/2 plus silica and 0.6 μM cyanocobalamin (Millipore‐Sigma) at the same temperature and light regime. *Chlamydomonas reinhardtii* UVM4 (Neupert *et al*., 2009) was grown in TAP without vitamins at 24°C and same light regime. Cultures were supplemented with thiamine (Acros Organics, Geel, Belgium), pyrithiamine (Sigma‐Aldrich), or 4‐amino‐5‐hydroxymethyl‐2‐methylpyrimidine (HMP; Sigma‐Aldrich), at the indicated concentrations for each of the experiments. Zeocin (InvivoGen, San Diego, CA, USA) at 75 mg l^−1^ was used to select transgenic *P. tricornutum* cells and at 10 mg l^−1^ to select for *C. reinhardtii* transformants. Cell growth was measured as optical density (OD)_730_ with a ClarioStar plate reader (BMG Labtech, Ortenberg, Germany).

### Prediction of TPP aptamers in newly sequenced diatom genomes

The sequence spanning from the 3′ strand of the P2 stem to the 3′ strand of the P4 stem of eight previously predicted TPP aptamers in diatoms (Croft *et al*., 2007; McRose *et al*., 2014; Supporting Information Table S1a) were used to create hidden Markov models (HMM) for both the forward and the reverse complement. The models were generated by multiple sequence alignments using Mafft (v.7.475; Katoh & Standley, 2013), followed by HMM model construction in Hmmer (v.3.1b2; Eddy, 2011). Both profiles were searched for against a custom sequence database of diatom genomes (Table S2) using Hmmer's ‘*hmmsearch*’ function with default parameters. Resulting hits were validated manually and their immediate upstream or downstream open reading frame was annotated through a Pfam database search and a reciprocal tBlastn with *P. tricornutum* (Table S1b). The secondary structure for each predicted aptamer associated with a thiamine‐related gene was annotated manually with the assistance of the RNAfold web server tool (Hofacker, 2003; Table S1c). PolyA signal site prediction on *PtTHIC* 3′UTR was performed with the Paspa server using default parameters (Ji *et al*., 2015).

### Identification of thiamine biosynthesis capacity in available diatom genomes

A Benchmarking Universal Single‐Copy Orthologue analysis (Busco, v.5.1.2) was performed using the genome mode to assess completeness of the various assemblies in our custom diatom genome database (Table S2; Seppey *et al*., 2019). Only assemblies with > 88% (stramenopiles_odb10) and 55.7% (eukaryota_odb10) complete Buscos were analysed further. Nucleotide assembly files from the resulting 19 diatom genomes were used to construct Blast databases. tBlastn searches (Blast+, v.2.6.0+) were performed for members of all KEGG orthologues associated with thiamine metabolism (KEGG:ko00730) using reference peptide sequences recovered from the KEGG database (Kanehisa & Goto, 2000; Kanehisa, 2019; Kanehisa *et al*., 2021) as queries (Table S3a). In addition, we also performed tBlastn searches with the predicted thiamine biosynthesis proteins THIC, TH1, THIS, THIO, THIG, THIF, DXS, TPK1, THI4, THIM, as well as the thiamine‐related proteins SSSP, SSUA/THI5‐like and TENA from *P. tricornutum* or *C. reinhardtii* (Table S3c). The best hit for each genome–protein pair was extracted, with full results reported in Table S3(b,d). For species with available annotation data, the overlap of tBlastn results with genomic loci was determined. In those cases in which annotation data were lacking or hits did not directly overlap with a genetic locus, contig/scaffold name together with start and end coordinates of the hit are provided. Categorising the presence of thiamine biosynthesis genes in a diatom genome was performed based on hits found by either one of the described tBlastn searches with an *E*‐value cut‐off of ≤10^−20^, while an *E*‐value between 10^−3^ and 10^−20^ indicated potential existence (Table S3e). Predicted peptide sequences containing NMT1 domains were analysed by multiple sequence alignments in Mega‐X v.10.1.1 (Kumar *et al*., 2018) using Muscle (Edgar, 2004) with default parameters and the phylogenetic tree was generated with the default Maximum‐Likelihood algorithm and 100 bootstrap iterations.

### RNA isolation and protein isolation

RNA was extracted from liquid nitrogen‐frozen cell pellets, prepared from 20 ml cultures grown to early stationary phase, using the RNeasy Plant Mini Kit (Qiagen). Immediately after extraction, the RNA samples were treated with 1 U of Turbo DNase (Thermo Fisher Scientific, Waltham, MA, USA) for 30 min before cDNA synthesis. Total protein extracts were obtained from 150 ml cultures grown to early stationary phase by resuspending in X ml of 0.2 M sorbitol (Sigma‐Aldrich), 1% β‐mercaptoethanol and 0.8 M Tris–HCl pH 8.3 (Sigma‐Aldrich), where X is equal to the culture OD_750_ before harvesting.

### Analysis of gene expression by quantitative PCR

First‐strand cDNA was generated using SuperScript III reverse transcriptase (Thermo Fisher Scientific) primed with random hexamers. Quantitative PCR was performed using SybrGreen JumpStart *Taq* (Sigma‐Aldrich) in a RotorGene qPCR thermocycler (Qiagen) for 40 cycles of 94°C for 20 s, 55°C for 20 s, and 72°C for 30 s (please refer to primers in Table S4). Total transcript levels of genes of interest were normalised to the levels of reference genes histone 4 (H4), ubiquitin conjugating enzyme (UBC) and ubiquitin (UBQ). Relative expression was calculated using the ΔΔ*C*_t_ method adjusted by amplification efficiency. Measurements with amplification efficiencies < 1.525 (1.67 for cobalamin supplementation experiment) were excluded. Reference genes showing significant differences between treatments were not used for normalisation.

### 3′ Rapid amplification of cDNA ends (RACE)

First‐strand cDNA was synthesised using a polyT‐VN primer with two anchor nucleotides at its 3′ end and a universal adaptor in its 5′UTR (Table S4) (Beilharz & Preiss, 2009). The cDNA was diluted 1 : 8 in nuclease‐free water and used as template for a first touch‐down RT‐PCR reaction (15 s annealing: 5 cycles 72°C, 5 cycles 70°C, 5 cycles 68°C, 20 cycles 65°C; 30 s extension) primed with a gene‐specific primer (71°C annealing *T*_m_) and a universal reverse primer using Q5 High Fidelity polymerase (New England Biolabs, Hitchin, UK). The PCR product of this first RT‐PCR was then diluted 1 : 100 in nuclease‐free water and used as a template for a seminested RT‐PCR using a gene‐specific primer and the universal reverse primer. Q5 polymerase was used again for 35 cycles using an annealing temperature of 65°C and 30 s extension. RT‐PCR products were run on a 2% agarose gel at 130 mV for 25 min unless otherwise stated. Selected bands were cut, purified with the Illustra™ GFX™ PCR DNA and Gel Band Purification Kit (Sigma‐Aldrich) and sent for Sanger sequencing (Source Bioscience UK Ltd, Notthingham, UK).

### Plasmid construction and algae transformation

All constructs were generated following the MoClo Golden Gate cloning approach (Engler *et al*., 2014). Level 0 parts were reused from existing *P. tricornutum* constructs from the *C. reinhardtii* MoClo Kit (Crozet *et al*., 2018) or were amplified from *P. tricornutum* genomic DNA using Q5 High Fidelity polymerase (please refer to Table S4 for primers). Level 1 constructs were assembled using *Bsa*I restriction ligation of Level 0 parts. Level 2 constructs were assembled by *Bpi*I restriction ligation of Level 1 constructs. Constructs used for CRISPR/Cas9 genome editing were generated following the sgRNA design strategy described in Hopes *et al*. (2017) and homologous recombination regions were designed to be *c*. 800‐bp long and flank the coding sequence of the gene of interest. The level 1 plasmid encoding a Cas9‐YFP expression cassette (pICH47742:PtFCP:Cas9YFP), the level 0 plasmid containing the *PtU6* promoter to drive expression of the sgRNAs (pCR8/GW:PtU6) and the plasmid used as template to amplify the sgRNA scaffold (pICH86966::AtU6p::sgRNA_PDS) were kind gifts from Dr Amanda Hopes and Professor Thomas Mock (UEA, Norwich, UK) and are available on Addgene (Hopes *et al*., 2016).

*Chlamydomonas reinhardtii* transformation was carried out as described in Mehrshahi *et al*. (2020) and *P. tricornutum* transformation was followed as in Yu *et al*. (2021). For co‐transformation of plasmids in *P. tricornutum*, 2.5 μg of each plasmid was used. For each construct, up to 96 primary zeocin‐resistant transformants were initially selected for PCR genotyping and preliminary phenotyping, and then a subset was taken for further characterisation.

### Determination of intracellular vitamin quotas

Cell pellets were harvested from 30‐ml cultures 5 d post‐inoculation, washed three times with distilled water, and fresh weight of the final pellets was measured before flash freezing in liquid nitrogen and storing at −80°C. Pellets were treated with 250 μl 1% (v/v) trichloroacetic acid (TCA) (Sigma‐Aldrich) and centrifuged at 10 000 ***g*** for 10 min recovering the supernatant. Thiamine pyrophosphate and thiamine were then derivatised by mixing 50 μl of the cell extract with 10 μl of freshly prepared 30 mM potassium ferricyanide (Sigma‐Aldrich) in 15% (w/v) sodium hydroxide, 15 μl 1 M NaOH and 25 μl methanol (high performance liquid chromatography (HPLC)‐grade; Sigma‐Aldrich). The derivatisation mix was centrifuged at 4000 ***g*** for 10 min and 20 μl of the supernatant were injected for HPLC analysis. An Accela HPLC setup (Thermo Fisher Scientific) was used equipped with a C18 150 × 4.6 mm column (Phenomenex, Macclesfield, UK). The fluid phase flowed at 1 ml min^−1^ with a gradient of 5% methanol up to 47.5% at 10 min, 100% at 11 min, 100% at 15 min, 5% at 16 min and equilibration at 5% methanol until 21 min. The thiamine and TPP derivatives were measured using a Dionex UltiMate 3000 fluorescence detector (Thermo Fisher Scientific) with 375 nm excitation and 450 nm emission. The sensitivity of the fluorescence detector was set at 1 for the first 5 min of the HPLC programme and increased to 8 for the rest of the programme. The area of the first half (from onset to the maximum fluorescence) of the peak at 1.35 min and the area of the peak at 2.1 min were used to calculate the amount of TPP and thiamine respectively relative to their respective standard curves.

### Western blots

Crude protein extracts were mixed with 1% sodium dodecyl sulphate (SDS; Sigma‐Aldrich) and boiled for 1 min. The samples were centrifuged at 16 000 ***g*** for 2 min, and 15 μl were analysed by SDS–polyacrylamide gel electrophoresis (SDS‐PAGE; 15% acrylamide). The electrophoresis was run at 150 mV for 90 min. The proteins were then transferred to a polyvinylidene difluoride (PVDF) membrane applying 20 mA for 20 min in a semidry transfer cell (Bio‐Rad Laboratories, Watford, UK). The membrane was blocked in 0.5% powdered milk in Tris‐buffered saline‐Tween (TBS‐T) buffer at 4°C overnight, then incubated for 1 h with a rabbit anti‐HA primary antibody (H6908; Sigma‐Aldrich) in 2.5% powdered milk in TBS‐T, washed four times with TBS‐T, then incubated for 1 h with a goat antirabbit secondary antibody conjugated with a Dy800 fluorophore (SA5‐35571; Thermo Fisher Scientific) in 2.5% powdered milk in TBS‐T. The membrane was finally washed four times in TBS‐T and once in TBS before being imaged in a fluorescence scanner (Odyssey; Li‐Cor Biotechnology, Cambridge, UK).

## Results

### Putative TPP aptamers can be found with high conservation in diatom genomes

To analyse the conservation and prevalence of putative TPP aptamers in diatoms, we searched for them in 23 available diatom genomes that were well assembled and annotated (Table S2). We performed HMM searches with a motif based on eight previously predicted diatom TPP aptamer sequences in *P. tricornutum*, *T. pseudonana*, *F. cylindrus* and *P. multiseries* (Croft *et al*., 2007; McRose *et al*., 2014; Table S1a). We found a total of 40 new putative TPP aptamers (Table S1b). An additional, more targeted, search for the universally conserved ‘CUGAGA’ motif in the UTRs of annotated *THIC* and *SSSP* genes revealed an additional putative TPP aptamer in the 3′UTR of *Psammoneis japonica THIC* that had not been detected by the HMM motif search.

**Fig. 1:**
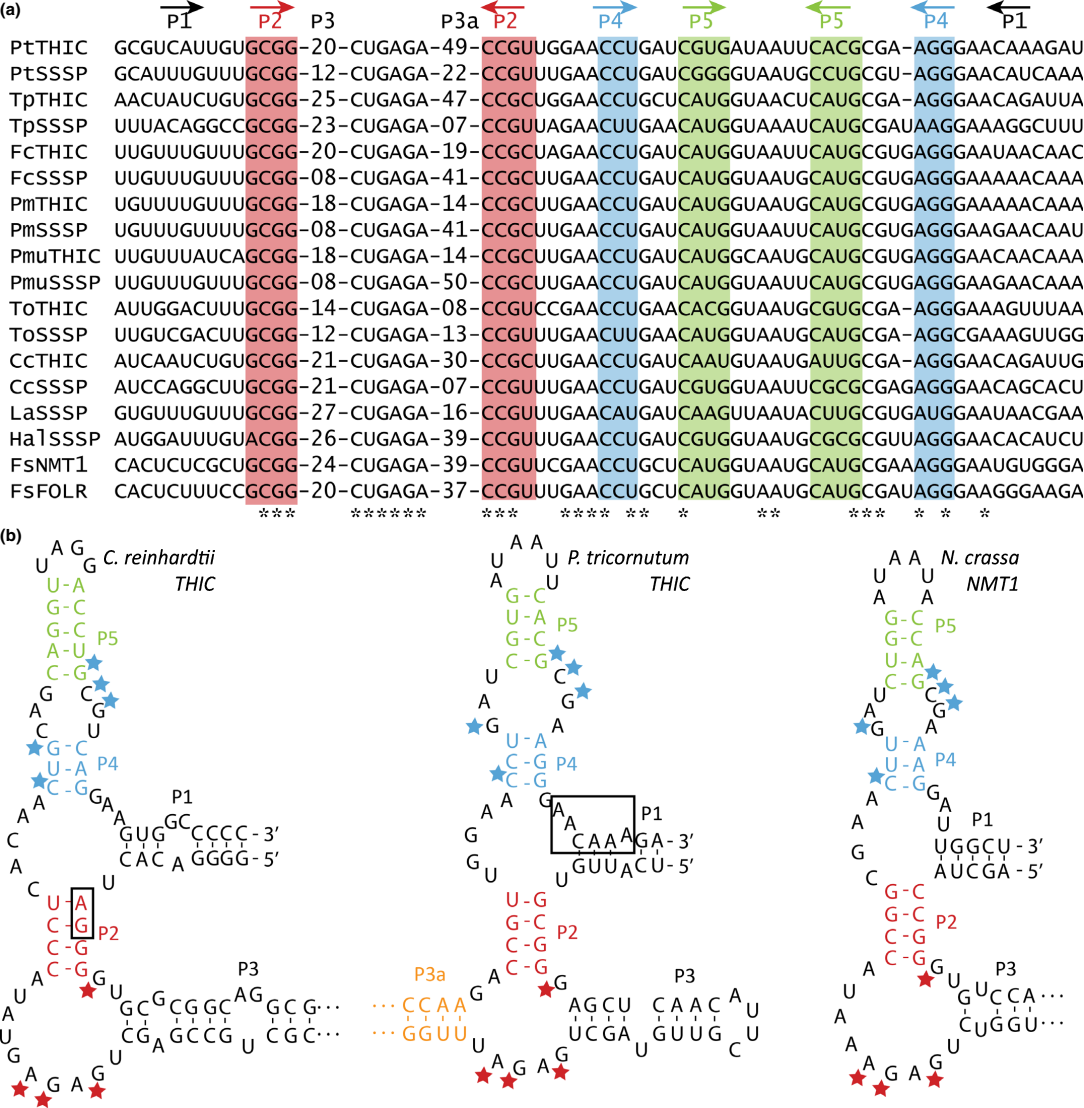
Multiple sequence alignment of 16 predicted diatom thiamine pyrophosphate (TPP) aptamers and structural comparison with previously characterised eukaryotic riboswitches. (a) Multiple sequence alignment of previously identified (first eight) and a sample of newly identified TPP aptamers in diatoms. Stems are indicated with arrows and are colour coded, asterisks indicate conserved residues across all sequences presented. See Supporting Information Table S1(b,c) for the full sequences of all predicted TPP aptamers. (b) Structural comparison of the predicted *Phaeodactylum tricornutum* HMP‐P synthase (THIC) aptamer (centre) with experimentally described TPP aptamers in *Chlamydomonas reinhardtii* (left; Croft *et al*., 2007) and *Neurospora crassa* (right; Cheah *et al*., 2007). The pyrimidine‐binding residues (‘CUGAGA’ motif, red stars) and the pyrophosphate‐binding residues (‘GCG’ motif, blue stars) are highlighted. Green algae and plant aptamers contain an alternative 3′ splicing site used in their mechanisms of action in their P2 stem (AG, boxed). The ‘AACAAA’ sequence overlapping with the *PtTHIC* aptamer P1 stem (boxed) was predicted by Paspa software to be the most likely polyadenylation site (Ji *et al*., 2015). Cc, *Cyclotella cryptica*; Fc, *Fragilariopsis cylindrus*; Fs, *Fistulifera solaris*; Hal, *Halamphora* sp. *MG8b*; La, *Licmophora abbreviata*; Pm, *Pseudonitzschia multiseries*; Pmu, *Pseudonitzschia multistriata*; Pt, *Phaeodactylum tricornutum*; To, *Thalassiosira oceanica*; Tp, *Thalassiosira pseudonana*.

All putative diatom TPP aptamers were found in 3′UTRs and shared a strong sequence conservation of the P2, P4 and P5 stems as well as a structurally conserved P3a stem of variable length (Fig. 1a; Table S1c). The P1 stems at the 3′ end of the putative aptamers are generally A‐rich and, in *PtTHIC* (*Phatr3_J38085*), the P1 stem overlaps with the ‘AACAAA’ motif that has been predicted to be the most likely polyadenylation site in the gene 3′UTR using Paspa software (Ji *et al*., 2015; Fig. 1b). The ‘CUGAGA’ motif and overall secondary structure architecture was conserved between diatoms and other aptamers demonstrated to be functional in green algae (Croft *et al*., 2007), plants (Wachter *et al*., 2007) and fungi (Cheah *et al*., 2007) (Fig. 1b). The P4/5 stem sequence was also well conserved between aptamers from the different groups. By contrast, the P2 stem differed between diatoms and other characterised TPP riboswitches. Interestingly, in green algae and plants, the P2 stem includes the ‘AGGG’ sequence, which contains the alternative splicing acceptor (AG) used in the mechanism of action determined experimentally (Croft *et al*., 2007; Wachter *et al*., 2007); in contrast, diatoms had a P2 stem with a conserved ‘GCGG’ sequence, with no obvious AG splicing acceptor nearby in the aptamer.

**Fig. 2:**
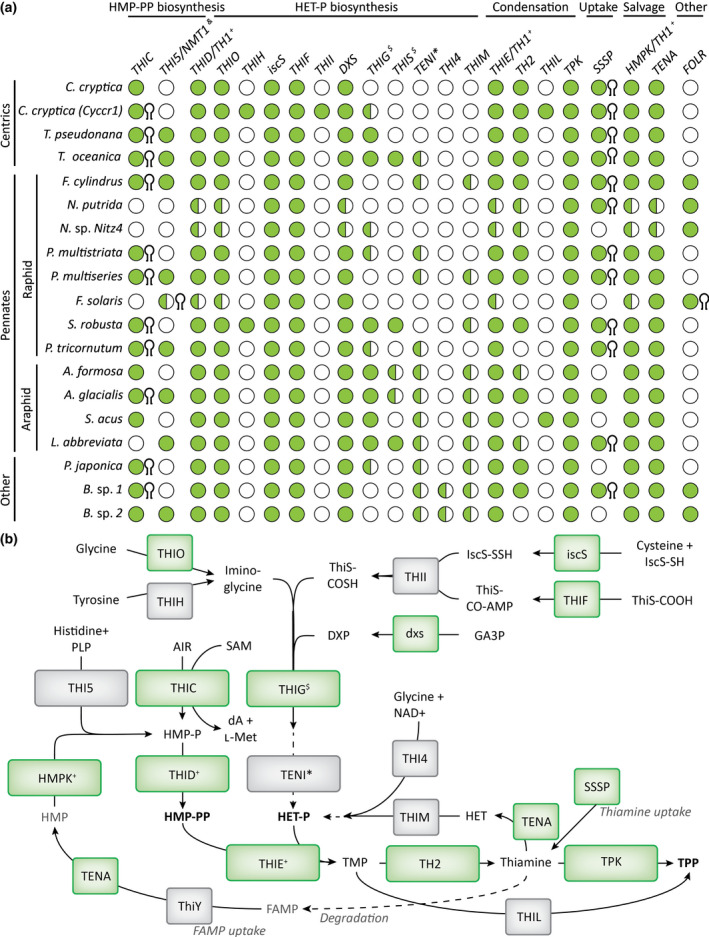
Proposed routes for thiamine biosynthesis in diatoms. (a) A tBlastn search using selected algal peptide sequences as queries (please refer to Supporting Information Table S3c) was performed against 19 diatom genomes to determine the presence (full circle *P*‐value > 10^−20^; half‐full circle *P*‐value > 10^−3^) or absence (empty circle) of different thiamine‐related genes. The presence of an associated predicted riboswitch in the 3′UTR of the gene is indicated with a hairpin symbol at the right of the circle. The genome abbreviations, accession numbers and references can be found in Table S2. (b) Potential thiamine biosynthetic, salvage and uptake routes in diatoms. The pathway steps with strong support across the diatom lineage are shown in green. AIR, 5‐aminoimidazole ribotide; dA, 5′‐deoxyadenosine; DXP, 1‐deoxy‐d‐xylulose 5‐phosphate; FAMP, *N*‐formyl‐4‐amino‐5‐aminomethyl‐2‐methylpyrimidine; GA3P, glyceraldehyde 3‐phosphate; HET‐P, hydroxyethyl‐thiazole phosphate; HMP‐P, hydroxymethyl‐pyrimidine phosphate; HMP‐PP, hydroxymethyl‐pyrimidine pyrophosphate; l‐Met, l‐methionine; NAD, nicotinamide adenine dinucleotide; PLP, pyridoxal 5′‐phosphate; SAM, *S*‐adenosyl methionine; TMP, thiamine monophosphate; TPP, thiamine pyrophosphate. ^&^ *THI5/NMT1* candidates contain an NMT1 Pfam domain (PF09084). ^$^ *THIG* and *THIS* are encoded in the chloroplast in *Phaeodactylum tricornutum*, so the results can be biased in genomes that do not include chloroplast sequences. ^+^THID, THIE and HMPK functions are performed by a single peptide in diatoms (TH1). *In some bacteria TenI accelerates a thiazole tautomerisation reaction, but it is not necessary to synthesise HET‐P (Hazra *et al*., 2011).

Thirty‐one (78%) of the newly predicted aptamers were directly associable with a potential genetic locus involved in thiamine metabolism, predominantly *THIC* and *SSSP*. Overall, TPP aptamers were associated with 11 of the 15 identified *THIC* genes and 12 of the 13 identified *SSSP* genes (Fig. 2a). In addition, putative TPP aptamers were found in genes encoding FOLR domains (folate receptor domain, PF03024) in *Halamphora* sp. MG8b and *Fistulifera solaris*. Proteins with FOLR domains in *F. cylindrus*, *Nitzschia* sp. Nitz4 and *Bacillariophyta* sp. (ASM1036717v1), but not in *F. solaris*, were predicted to have a signal peptide using SignalP 4.1, suggesting a potential role in transport or sensing. Predicted TPP aptamers were also found to be associated with multiple copies of *F. solaris* genes encoding an NMT1 domain (PF09084; No Message in Thiamine; Maundrell, 1990) (Fig. 2a; Table S2c). The bioinformatics analysis also provided the means to construct the complete pathway of thiamine metabolism in diatoms, indicating both the biosynthetic and salvage routes for provision of the active co‐factor, TPP (Fig. 2b). This confirms that synthesis of the pyrimidine moiety uses THIC, as in plants and green algae (as well as bacteria), but that the thiazole group is synthesised via the THIG bacterial route, rather than THI4/THI1 as in all other eukaryotes analysed to date.

In addition to THIC, nine out of 19 diatom genomes queried revealed at least one gene that encoded a protein with an NMT1 domain. These were associated with THI5, an HMP‐P synthase in fungi, but they also have structural homology with ThiY, a bacterial periplasmic component of a pyrimidine precursor ABC transporter (Bale *et al*., 2010). To investigate whether the diatom candidates with NMT1 domains showed closer similarity to THI5 or ThiY, we aligned 10 diatom protein sequences with NMT1 domains with *Bacillus halodurans* ThiY, *Saccharomyces cerevisiae* THI5, *Neurospora crassa* NMT1 and peptide sequences with NMT1 domains previously identified in other algal species (McRose *et al*., 2014). Multiple sequence alignment and subsequent phylogenetic tree analysis showed that the diatom candidates clustered with the haptophyte (*Emiliana huxleyi*) and cryptophyte (*Guillardia theta*) candidates in a single branch, except for two candidates found in *F. solaris*, which clustered with the chlorophyte peptides (Fig. S1a). However, the phylogenetic analysis failed to resolve whether the algal proteins containing NMT1 domains were more closely related to THI5 or ThiY, with bootstrap values below 60. The multiple sequence alignment also revealed that the diatom candidates conserved only four of the 15 active site residues in THI5, and four out of eight active site residues in ThiY (Fig. S1b). Additionally, except in *F. solaris*, all diatom candidates show an extended N‐terminus as in ThiY, which was predicted to be a signal peptide using SignalP v.4.1 (Petersen *et al*., 2011).

To test whether diatom candidates with NMT1 domains were expressed and regulated by its putative metabolic products, we used RT‐qPCR to measure the transcript levels of the *P. tricornutum* and *T. pseudonana* candidates (*Phatr3_J33535* and *THAPS_6708* respectively) in the presence or absence of thiamine or HMP supplementation. The results confirmed that the candidates were expressed in both species, but they were not regulated by thiamine (Fig. S2).

### *THIC* transcript levels are unaffected by exogenous thiamine and *P. tricornutum* and *T. pseudonana* are resistant to pyrithiamine

**Fig. 3:**
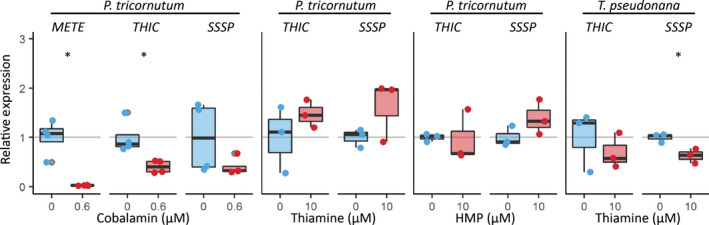
Impact of vitamin supplementation on expression of *THIC* and *SSSP* in *Phaeodactylum tricornutum* and *Thalassiosira pseudonana*. *P. tricornutum* and *T. pseudonana* were grown in the absence (blue) or presence (red) of 0.6 μM cobalamin (B_12_), 10 μM thiamine (B_1_) or 10 μM 4‐amino‐5‐hydroxymethyl‐2‐methylpyrimidine (HMP) for 7 d. Three or four biological replicates were analysed by RT‐qPCR in technical duplicate. The technical replicate measurements were averaged for each biological replicate, and transcript levels were normalised for the average transcript levels of three housekeeping genes (*H4*, *UBC*, *UBQ* for *P. tricornutum*; *Actin*, *EF1a*, *rbcs* for *T. pseudonana*). Each dot represents the relative expression value for an individual biological replicate and a box plot summarises the data for each gene and treatment. Two‐sided *t*‐tests between supplemented and control conditions were conducted for all genes. *, *P*‐value < 0.05.

To investigate whether putative riboswitches in other thiamine‐related genes in *P. tricornutum* and *T. pseudonana* responded to exogenous thiamine, an RT‐qPCR experiment was carried out with cells grown in the presence or absence of 10 μM thiamine or 10 μM HMP, both of which reduced the expression of *THIC* in *C. reinhardtii* (Moulin *et al*., 2013). A previous transcriptomics and proteomics study (Bertrand *et al*., 2012) had shown that *PtTHIC* was affected by growth of cells in cobalamin (vitamin B_12_), so this was also included. As expected, in *P. tricornutum*, *PtTHIC* levels dropped approximately two‐thirds (*P*‐value 0.03) in the presence of cobalamin relative to the unsupplemented condition (Fig. 3). The positive control, *PtMETE* (Helliwell *et al*., 2011), showed a 97% reduction (*P*‐value 0.01). By contrast, neither thiamine or HMP supplementation caused significant changes in transcript levels of *PtTHIC* or *PtSSSP* (*Phatr3_J50012*). Similarly in *T. pseudonana*, *TpTHIC* (*THAPSDRAFT_41733*) transcript levels were unaffected when cells were cultured with 10 μM thiamine. However, in contrast with *P. tricornutum*, thiamine supplementation resulted in an approximately one‐third downregulation (*P*‐value 0.03) of *TpSSSP* (*THAPSDRAFT_20656*). To rule out the possibility that these results were explained by the inability of exogenous thiamine to enter the cells, we performed a thiamine uptake test and confirmed that thiamine was actively taken up and metabolised to TPP in *P. tricornutum* (Fig. S3).

Acknowledging that gene regulation could also happen post‐transcriptionally and given the presence of a predicted polyadenylation site overlapping the P1 stem in *PtTHIC*, we used a 3′RACE experiment to test whether the putative *PtTHIC* aptamer could regulate gene expression via alternative polyadenylation or alternative splicing. The results showed no substantive difference in *PtTHIC* 3′UTR isoforms between the control and the thiamine or HMP‐supplemented conditions (Fig. S4). These results suggested that the *PtTHIC* predicted riboswitch did not regulate expression at a transcriptional or post‐transcriptional level in response to thiamine.

**Fig. 4:**
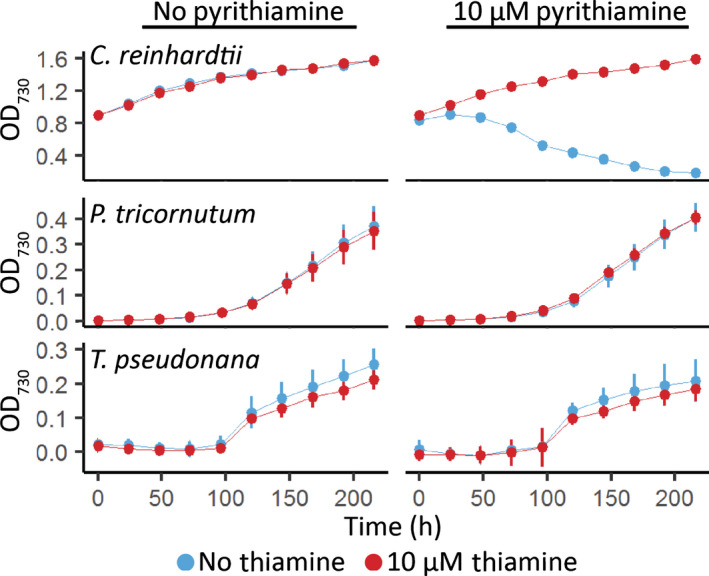
Effect of the thiamine antimetabolite pyrithiamine on the growth of *Chlamydomonas reinhardtii*, *Phaeodactylum tricornutum* and *Thalassiosira pseudonana*. *C. reinhardtii*, *P. tricornutum* and *T. pseudonana* were grown for 9 d in the absence (left column) or presence (right column) of 10 μM pyrithiamine and the absence (blue) or presence (red) of 10 μM thiamine in 96‐well plates. Growth was measured as optical density (OD)_730_ every 24 h. Error bars represent the standard deviation of three biological replicates.

Finally, to test experimentally whether the putative diatom aptamers could regulate thiamine metabolism, we used a pyrithiamine growth assay, previously used to study thiamine gene regulation in other organisms (Sudarsan *et al*., 2005). Briefly, pyrithiamine, a thiamine antimetabolite, binds to the TPP aptamer, downregulating the expression of thiamine biosynthesis genes regulated by TPP riboswitches, preventing the production of thiamine, and inducing growth arrest. The lethal effect of pyrithiamine can be reversed by adding extracellular thiamine to compensate for the lack of biosynthetic activity. In this study, *C. reinhardtii*, *P. tricornutum* and *T. pseudonana* were grown in the presence or absence of 10 μM pyrithiamine and/or 10 μM thiamine (Fig. 4). As can be seen clearly, *C. reinhardtii* growth was disrupted by pyrithiamine and rescued by thiamine supplementation, but *P. tricornutum* and *T. pseudonana* were insensitive to the antimetabolite.

### The *P. tricornutum THIC* 3′UTR cannot regulate the expression of reporter constructs

**Fig. 5:**
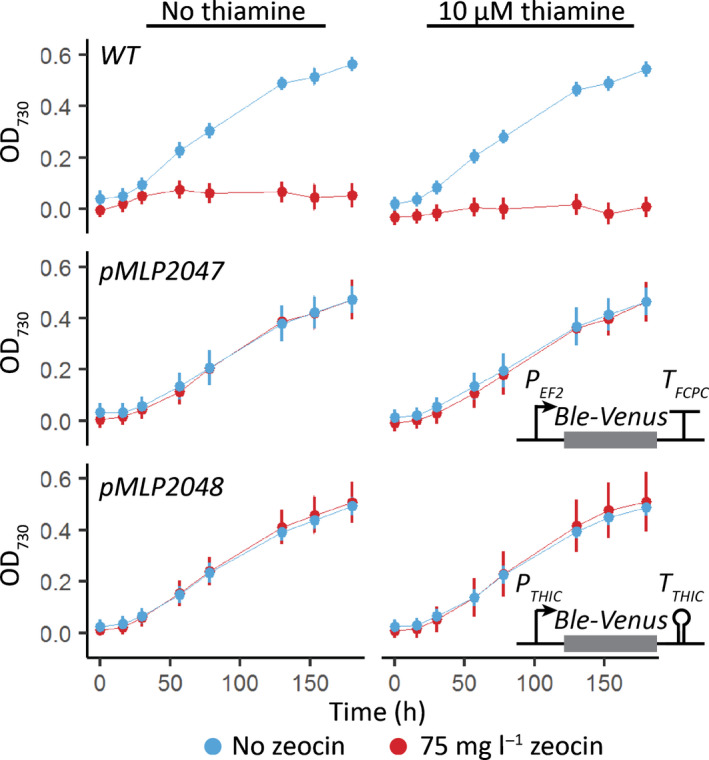
Effect of thiamine supplementation on transformants with *PtTHIC* promoter and 3′UTR driving expression of the *Ble* zeocin resistance gene. Transformants carrying a *Ble‐Venus* reporter controlled by the *PtEF2* promoter and *PtFCPC* 3′UTR (pMLP2047) or the *PtTHIC* promoter and 3′UTR (pMLP2048) were grown in the absence (left column) or presence (right column) of 10 μM and thiamine the absence (blue) or presence (red) of 75 mg l^−1^ zeocin. Error bars represent the standard deviation of three biological replicates for each of 10 independent transformants for pMLP2047 and pMLP2048 and three biological replicates for wild‐type (WT).

As an alternative approach to determine whether the putative TPP aptamers in diatoms could regulate expression in response to thiamine supplementation, we generated and utilised a set of constructs in which the putative *PtTHIC* riboswitch would regulate the expression of a reporter gene. We cloned the *PtTHIC* promoter, 5′UTR and 3′UTR so that they flanked a *Ble‐Venus* reporter gene that conferred resistance to zeocin. In principle, if the putative *PtTHIC* riboswitch regulated gene expression, the combined supplementation of thiamine and zeocin would induce a downregulation of the antibiotic‐resistance reporter gene, which would in turn lead to growth arrest. However, we could not see any impact on growth when the transformants were cultured in the presence of 10 μM thiamine and 75 mg l^−1^ zeocin, providing further evidence that the putative *PtTHIC* riboswitch did not respond to thiamine supplementation (Fig. 5).

**Fig. 6:**
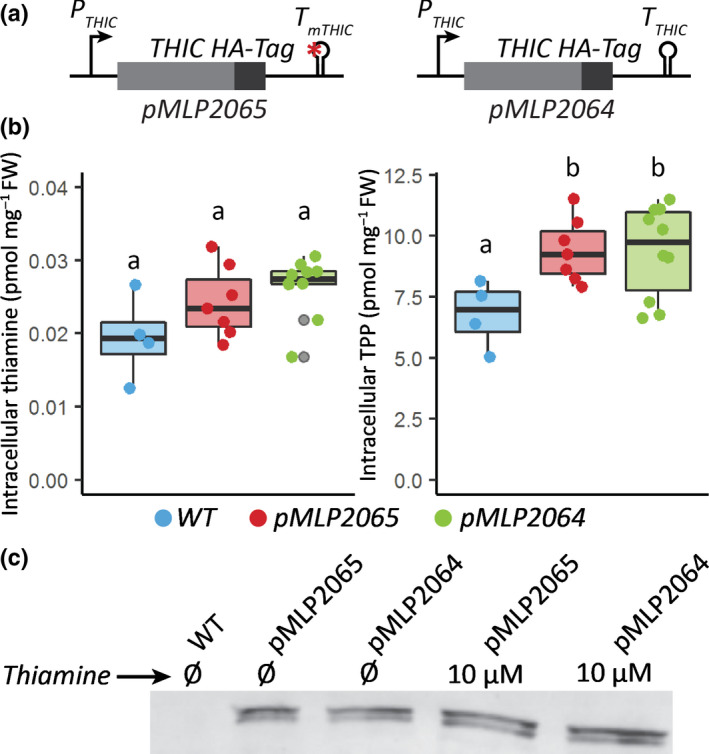
Intracellular thiamine and thiamine pyrophosphate (TPP) abundance and PtTHIC protein levels determined in transformants carrying a mutated *PtTHIC* aptamer. (a) A construct coding for an extra copy of *PtTHIC* with a targeted mutation in its putative aptamer (pMLP2065) and its respective unmutated control (pMLP2064) were transformed into *Phaeodactylum tricornutum*. (b) Transformants were grown for 5 d before thiamine and TPP were quantified by high performance liquid chromatography (HPLC) and normalised by fresh weight. Each dot represents the measurement of an independent transformant and a box plot summarises the data. Different letters represent significant differences in average vitamin content between strains in a Tukey honestly significant difference (HSD) test with a 0.95 confidence level. (c) An independent transformant for each construct was grown to *c*. 5 × 10^6^ cells ml^−1^ in the presence or absence of 10 μM thiamine, and protein was extracted from 150 ml cultures. A western blot analysis with a primary anti‐HA antibody on total crude extracts normalised to culture optical density (OD) is shown.

*Phaeodactylum tricornutum* is unlikely to encounter thiamine concentrations at the micromolar level in oceanic environments in which thiamine concentrations have been measured in the picomolar range (Sañudo‐Wilhelmy *et al*., 2012; Monteverde *et al*., 2015). Therefore, we wanted to test whether, despite being unresponsive to high levels of exogenous thiamine, the putative *PtTHIC* riboswitch was responsible for the homeostasis of intracellular TPP concentrations. To address this question, we used a mutational approach inspired by previous observations in *Arabidopsis thaliana* and *C. reinhardtii*, for which mutations affecting functional TPP riboswitches in thiamine biosynthetic genes led to the overaccumulation of thiamine and TPP in response to a disruption of the negative feedback regulatory mechanism (Bocobza *et al*., 2013; Moulin *et al*., 2013 respectively). To replicate these experiments in *P. tricornutum*, we transformed wild‐type (WT) cells with an extra copy of *PtTHIC* with a targeted mutation in the universally conserved pyrimidine‐binding motif of the putative aptamer (‘CUGAGA’ to ‘CUCUCU’). To generate a control strain, a construct without this mutation was also transformed (Fig. 6a). We then grew the transformants alongside a WT strain in the absence of exogenous thiamine for 5 d and quantified their intracellular thiamine and TPP levels by HPLC. Higher intracellular TPP levels for the transformants with a mutated or an unmutated extra copy of *PtTHIC* compared with WT indicated that the overexpression level was sufficient to cause an increase in metabolic flux. However, there was no significant difference in intracellular thiamine or TPP levels between the strains with the mutated copy of *PtTHIC* compared with the unmutated control, suggesting that the putative *PtTHIC* aptamer was not required to regulate the homeostasis of thiamine and TPP levels (Fig. 6b). In addition, the heterologous copies of *PtTHIC* in both constructs were tagged with a C‐terminal HA‐Tag so that we could follow changes in protein levels. If the riboswitch were functional, one would expect that a mutation in the universally conserved ‘CUGAGA’ motif would disrupt feedback regulation and lead to increased protein levels. Western blot assays of cell extracts showed no appreciable qualitative increase in heterologous PtTHIC protein levels between the mutated and control constructs (Fig. 6c). This was the case whether or not the cells were grown with exogenous thiamine, although as only one independent transformant for each construct was used, the results cannot be interpreted in a quantitative manner.

### The putative *PtTHIC* aptamer does not mediate switching in the *CrTHI4* 5′UTR aptamer platform

**Fig. 7:**
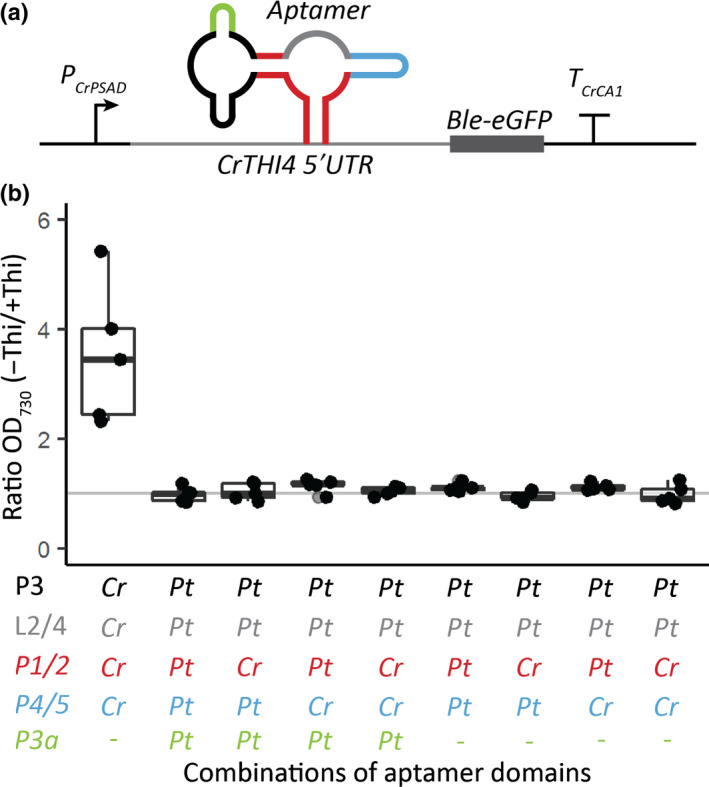
Response of *Chlamydomonas reinhardtii* carrying the *PtTHIC‐CrTHIC* chimeric riboswitches to thiamine supplementation. (a) The *CrTHI4* riboswitch platform previously developed in our laboratory (Mehrshahi *et al*., 2020) was cloned in the 5′UTR of a *Ble‐eGFP* zeocin resistance reporter with a *CrPSAD* promoter and a *CrCA1* terminator. A set of modified aptamers combining five structural parts (P1/2, P3, P3a, L2/4 and P4/5; colour coded) from *CrTHIC* and *PtTHIC* aptamers was cloned into the platform and the constructs transformed into *C. reinhardtii*. (b) Five independent transformants for each modified aptamer design were grown in the presence of 10 mg l^−1^ zeocin with or without 10 μM thiamine for 4 d. The ratio between the optical density (OD)_730_ in the depleted and the thiamine‐supplemented conditions is shown.

To test whether the putative *PtTHIC* aptamer was able to bind TPP and thereby regulate gene expression, we used an aptamer testing platform that we had recently developed in *C. reinhardtii* (Mehrshahi *et al*., 2020), which provides a simple measurable growth readout. Briefly, the aptamer platform allows the introduction of heterologous aptamers into a modified *CrTHI4* 5′UTR containing the riboswitch cloned in front of a *Ble‐eGFP* reporter (Fig. 7a). As before, if the introduced aptamers are functional in the platform context, the simultaneous presence of thiamine and zeocin in the medium impairs growth. In this study, we introduced the putative *PtTHIC* aptamer into the aptamer platform and used the *CrTHIC* aptamer as a positive control to test whether the putative *PtTHIC* aptamer could respond to thiamine. We found that, as seen previously, the transformants with the *CrTHIC* aptamer showed impaired growth in the presence of thiamine and zeocin, with over a three‐fold difference in OD_730_ between the thiamine‐depleted and ‐supplemented conditions 4 d post‐inoculation (Fig. 7b). By contrast, the transformants with the putative *PtTHIC* aptamer showed no growth difference between thiamine replete and deplete treatments. We then prepared a suite of modified aptamers combining functional domains from *CrTHIC* and *PtTHIC* aptamers to test whether a particular functional domain of the *PtTHIC* aptamer was responsible for the lack of thiamine response or was not compatible with the aptamer testing platform (Fig. 7a). We found that neither introducing the P1 and P2 stems and/or the P4/5 stem from *CrTHIC* aptamer into the *PtTHIC* aptamer nor removing the P3a stem led to a responsive aptamer. In one of the modified aptamers, the only difference from the *CrTHIC* positive control was the L2/4 loop and the P3 stem from the *PtTHIC* aptamer and yet this variant still failed to respond to thiamine (Fig. 7b).

### *P. tricornutum* *SSSP* is necessary for thiamine uptake and *THIC* is essential for thiamine biosynthesis

**Fig. 8:**
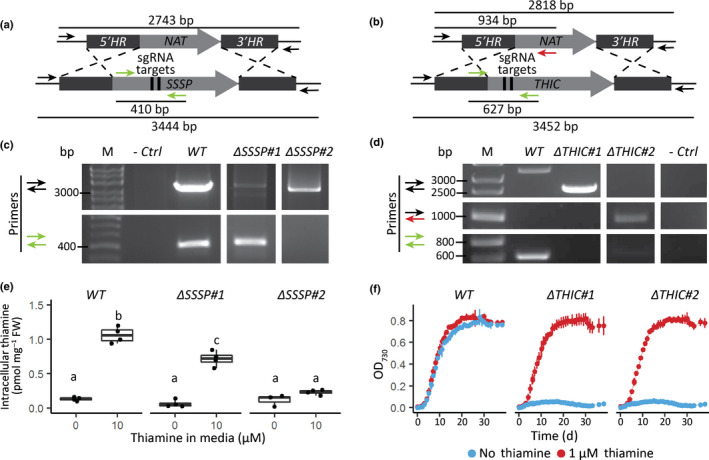
Determination of genotype and phenotype of *Phaeodactylum tricornutum SSSP* and *THIC* CRISPR/Cas9 mutants. (a, b) Schematic representation of the CRISPR‐mediated homology recombination strategy to inactivate *SSSP* and *THIC*, respectively. (c, d) Transformants were genotyped with two or three primer pairs colour coded in panel (a). The negative control did not include any template DNA. (e) Wild‐type (WT) and two *SSSP* knock‐out strains were grown in the absence or presence of 10 μM thiamine for 5 d in biological duplicate, and intracellular thiamine levels were measured in technical duplicate. Different letters represent significant differences in average intracellular thiamine content between strains and conditions in a Tukey honestly significant difference (HSD) test with a 0.95 confidence level. (f) WT and two *THIC* knock‐out strains were grown in the absence (blue) or presence (red) of 1 μM thiamine in 24‐well plates recording growth as OD_730_ every 24 h. Error bars represent the standard deviation of three biological replicates.

Having observed that *PtTHIC* is not regulated by thiamine, unlike its homologues in bacteria, plants and chlorophytes, we sought to investigate whether the two *P. tricornutum* genes with putative TPP aptamers, *PtSSSP* and *PtTHIC*, were genuinely involved in thiamine metabolism in this diatom. We used CRISPR/Cas9‐induced homologous directed repair to knock‐out the genes and study their function. We generated knock‐out strains for *PtTHIC* and *PtSSSP* by co‐electroporating WT cells with a plasmid coding for Cas9 and a guide RNA pair, and another plasmid encoding a homologous repair template designed to swap the coding sequence of each target gene for a nourseothricin resistance cassette. This design facilitates genotypic screening by PCR and phenotypic screening by nourseothricin resistance (Fig. 8a,d). After an initial screen of several hundred nourseothricin‐resistant transformants, we identified several transformants with insertions in the *PtSSSP* gene. Two were characterised further. Genotyping confirmed one mutant, called *ΔSSSP#1*, with a monoallelic disruption of the coding sequence around the sgRNA target sites, and a second mutant (*ΔSSSP#2*) with a biallelic disruption of the genomic sequence, in other words a complete knock‐out (Fig. 8b). When grown in the presence of 10 μM thiamine, intracellular thiamine levels in WT cells were substantially higher than the levels in the absence of the vitamin (1.06 pmol mg^−1^ fresh weight (FW) compared with 0.13 pmol mg^−1^ FW), whereas in the *ΔSSSP#1* mutant the increase in intracellular thiamine was only to 0.72 pmol mg^−1^ FW (Fig. 8c). There was no statistical difference in intracellular thiamine levels between *ΔSSSP#2* cells grown in the presence or absence of 10 μM thiamine (0.23 vs 0.09 pmol mg^−1^ FW) indicating that no exogenous thiamine had been taken up. This finding demonstrated that PtSSSP is essential for thiamine uptake and is likely to encode a thiamine transporter (Fig. 8c).

**Fig. 9:**
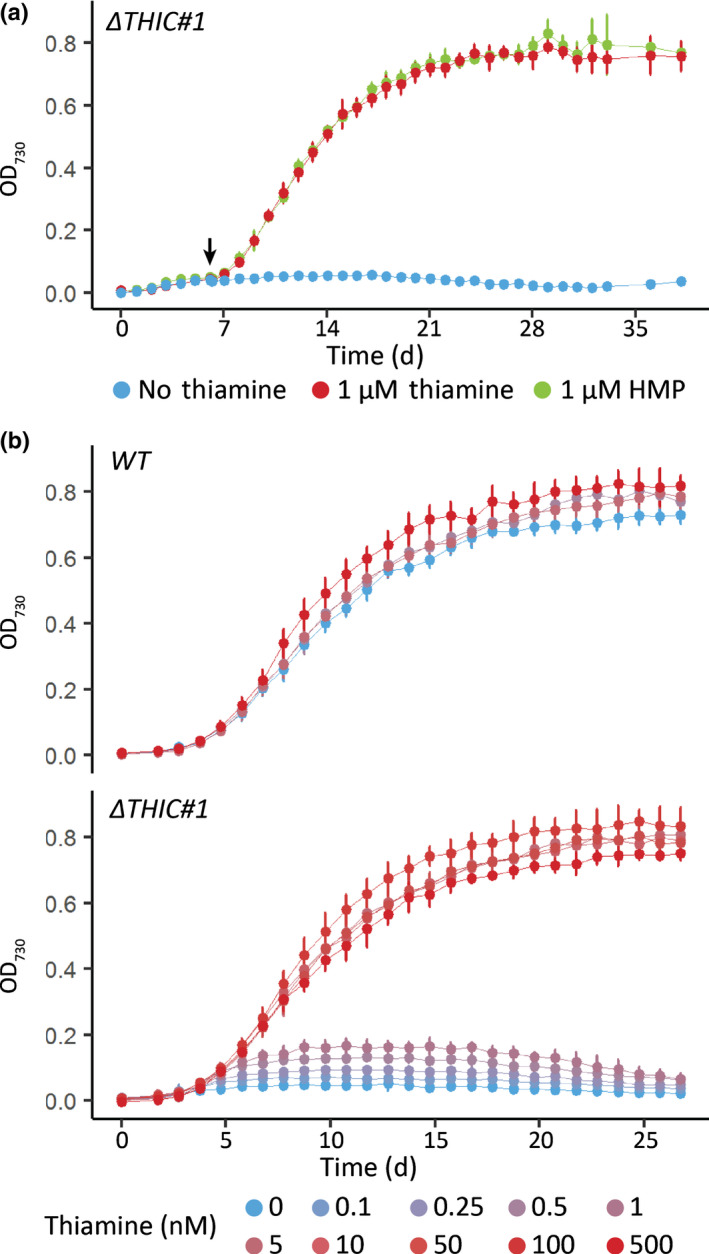
Phenotype analysis of a *PtTHIC* knock‐out mutant. (a) The *ΔTHIC#1* mutant was initially grown in the absence of supplementation, at day 6 (arrow) 1 μM thiamine (red) or 1 μM 4‐amino‐5‐hydroxymethyl‐2‐methylpyrimidine (HMP; green) was supplemented and growth compared with an unsupplemented control (blue). Error bars represent the standard deviation of three biological replicates. (b) Wild‐type (WT) and the *ΔTHIC#1* mutant were grown in increasing concentrations of thiamine (0–500 nM) to determine the thiamine concentration required to support growth of the mutant. Error bars represent the standard deviation of six biological replicates.

For *THIC*, again after an initial screen of hundreds of transformants from a CRISPR/Cas9 experiment, we characterised two of them further. Both independent mutants showed a biallelic loss of the *PtTHIC* coding sequence (CDS; Fig. 8e). Whereas thiamine supplementation (at 1 μM) had no effect on growth of a WT control, both *ΔTHIC* mutants were able to grow only in the presence of thiamine, with no growth observed in its absence (Fig. 8f). To confirm whether *PtTHIC* encoded an HMP‐P synthase, we started three cultures of the *ΔTHIC#1* mutant in the absence of thiamine and at day 6 post‐inoculation we supplemented the first culture with 1 μM thiamine, the second with 1 μM HMP, and the third was left unsupplemented. Both thiamine and HMP supplementation supported the growth of the mutant from that point (Fig. 9a), confirming that *PtTHIC* encoded an HMP‐P synthase. Finally, we grew the *ΔTHIC1* mutant in increasing concentrations of thiamine (0–500 nM) to establish the vitamin requirements of the mutant. As little as 5 nM thiamine was sufficient to support the growth of the mutant without detriment, but the mutant could not grow at 1 nM thiamine (Fig. 9b). In contrast, two independent biallelic knockout mutants of the *NMT1* gene, generated in the same way, showed no obvious phenotype compared with wild‐type and could grow in the absence of exogenous thiamine (Fig. S5).

## Discussion

Using a bioinformatics approach we screened over 20 published diatom genomes and found 41 previously unidentified putative TPP aptamer sequences, 32 of which were associated with four genes: *THIC*, *SSSP*, *SSUA/THI5‐like* (encoding NMT1 domains) and *FOLR* (Fig. 2; Table S1b). Riboswitches generally bind ligands related to the function of the genes with which they are physically associated (McCown *et al*., 2017), suggesting that *THIC*, *SSSP*, *SSUA/THI5‐like* and *FOLR* are involved in thiamine metabolism. We have been able to validate experimentally the function of the first two genes in *P. tricornutum*, and yet the putative TPP aptamer sequences do not operate as riboswitches.

The thiamine auxotrophy shown by the *P. tricornutum THIC* knock‐out mutants generated by CRISPR/Cas9 (Fig. 8f) demonstrated that diatom *THIC*s encode an HMP‐P synthase homologous to experimentally validated bacterial and plant HMP‐P synthases (Raschke *et al*., 2007; Chatterjee *et al*., 2008). Moreover, the inability of the *P. tricornutum ΔSSSP* mutant to uptake exogenous thiamine (Fig. 8c) demonstrated, for the first time in an algal species, that SSSP is required for thiamine uptake. SSSP belongs to the sodium‐solute transporter (SSS) family, often associated with B‐vitamin transporters (Jaehme & Slotboom, 2015). Members of this family have been found to be associated with predicted TPP riboswitches in chlorophytes, prasinophytes, cryptophytes, stramenopiles and haptophytes (McRose *et al*., 2014) but, to our knowledge, have never been validated experimentally before. SSSP is homologous to the experimentally confirmed thiamine transporter Dur31 in the fungus *Candida parapsilosis*, which is also associated with a TPP aptamer (Donovan *et al*., 2018).

We also identified candidate *SSUA/THI5‐like* genes containing NMT1 domains with homologies to the ThiY FAMP transporter (Bale *et al*., 2010) and the THI5 HMP‐P synthase (Coquille *et al*., 2012) in nine diatom genomes. Except for those in *F. solaris*, all diatom candidates, together with those in haptophytes and cryptophytes, had a conserved signal peptide, which is present in ThiY but not THI5. By contrast, the multiple copies of the *F. solaris* candidate are phylogenetically more closely related to those in prasinophytes and chlorophytes, in agreement with their suggested horizontal gene transfer origin (Vancaester *et al*., 2020), and the fact that they are associated with predicted TPP aptamers. None of the *F. solaris* candidates had the signal peptide present in ThiY and SSUA, and therefore it is unlikely that they have the same function. As the *P. tricornutum ΔTHIC* mutants cannot grow in the absence of thiamine (Fig. 8f), the NMT1 domain‐containing gene is not sufficient for the production of HMP‐P. Moreover, we observed that *P. tricornutum* CRISPR/Cas9 mutants lacking the NMT1 domain‐containing gene were able to grow in the absence of exogenous thiamine and did not show an observable phenotype compared with WT (Fig. S5), indicating that the NMT1‐containing gene is not necessary for the biosynthesis of thiamine. In addition, the haptophyte *E. huxleyi* has a NMT1 domain‐containing gene but lacks *THIC* and is unable to grow without thiamine or the HMP derivative 4‐amino‐5‐aminomethyl‐2‐methylpyrimidine (AmHMP; McRose *et al*., 2014; Gutowska *et al*., 2017). We propose that the previously used nomenclature ‘*SSUA/THI5‐like*’ (McRose *et al*., 2014) does not correspond to the actual function of NMT1 domain‐containing genes in diatoms and should not be used. Instead we hypothesise that (with the exception of *F. solaris*) they are related to the bacterial *ThiY* and so involved in the salvage of the pyrimidine moiety. Further auxotrophy tests with NMT1 domain‐containing gene mutants should be carried out to test conclusively whether these genes are involved in pyrimidine salvage. *FOLR* candidates were identified in six diatom genomes, and these are homologous to genes associated with predicted TPP aptamers in prasinophytes and rhizaria (McRose *et al*., 2014). Although the function of these genes remains unknown, they are predicted to have signal peptides, so they might be involved in thiamine transport through a receptor‐mediated endocytosis mechanism similar to the homologous folate (vitamin B_9_) receptors in mice and humans (Zhao & Goldman, 2013).

The strong sequence conservation between putative diatom TPP aptamers, particularly in their P2, P4 and P5 stems and in the TPP binding motifs (Fig. 1a), indicated that the sequences are likely to have retained a defined and shared function within the diatom lineage. Given the absence of a conserved splicing acceptor site in P2 stems and the lack of evidence for alternative splicing in the *PtTHIC* 3′UTR in previous transcriptomic studies (Maheswari *et al*., 2009), our initial hypothesis was that the predicted diatom TPP riboswitch mechanism involved alternative polyadenylation in contrast with the alternative splicing mechanism shown for all previously characterised eukaryotic TPP riboswitches (Nguyen *et al*., 2016). This hypothesis was further supported by the conservation of A‐rich P1 stems and the prediction of a polyadenylation site overlapping the *PtTHIC* aptamer P1 stem. In many eukaryotic genes, alternative polyadenylation determines differences in protein abundance, protein localisation and/or protein–protein interactions between different transcript isoforms via the inclusion or exclusion of *cis*‐regulatory elements bound by RNA‐binding proteins (Mayr, 2019).

However, despite the strong sequence conservation with experimentally characterised eukaryotic riboswitches and despite active thiamine uptake in *P. tricornutum* (Figs 8c, S3), we were not able to demonstrate a change in transcript levels, nor alternative splicing or alternative polyadenylation in *PtTHIC* 3′UTR in response to thiamine supplementation (Figs 3, S4). The failure of *PtTHIC* 3′UTR to regulate a zeocin resistance reporter (Fig. 5) and of the predicted aptamer to mediate a response to thiamine in the *CrTHI4* aptamer platform (Fig. 7) supported these observations and led us to conclude that the predicted *PtTHIC* riboswitch did not regulate gene expression in response to thiamine supplementation. The stable levels of intracellular thiamine and TPP in transformants carrying a mutated *PtTHIC* aptamer (Fig. 6) further demonstrated that under laboratory conditions the predicted *PtTHIC* riboswitch is also not necessary to regulate the homeostasis of intracellular thiamine levels. Although the RT‐qPCR results in *T. pseudonana* showed a one‐third downregulation of *TpSSSP* this change was only supported by a *P*‐value of 0.03, and *TpTHIC* levels did not respond to thiamine (Fig. 3). These results, together with the *P. tricornutum* and *T. pseudonana* resistance to pyrithiamine (Fig. 4), suggested that the lack of response to thiamine supplementation by the predicted TPP riboswitches could be shared throughout the diatom lineage. However, there is the possibility that differences in pyrithiamine uptake between *C. reinhardtii* and diatoms explained the differences in growth. Additionally, it is worth noting that HMP can be obtained from the degradation of pyrithiamine (Sudarsan *et al*., 2005) and if the thiazole biosynthetic pathway is unaffected, the organisms would be able to survive despite THIC downregulation by pyrithiamine. We have not predicted any TPP riboswitches associated with the thiazole biosynthetic pathway in diatoms, therefore the salvage of HMP could mask the results of the pyrithiamine experiment.

While all our experimental data coherently demonstrated that the predicted *PtTHIC* riboswitch does not respond to thiamine supplementation, it remains to be investigated whether it is the failure of the predicted diatom riboswitches to bind TPP or the failure to respond that explains the results shown here. RNA structure probing or equilibrium dialysis in the presence or absence of TPP could be used in the future to resolve these possibilities. Finally, the question remains why there is such sequence conservation across diatom aptamers, especially as these are in an untranslated region of the transcript. In general terms, we propose that the riboswitch studied here may have lost the function of regulating gene expression in response to thiamine supplementation. However, the sequence must still have a conserved function able to sustain strong selection pressure to conserve the sequence. Taken together, our results showed the weakness of bioinformatics approaches to predict riboswitch function and stressed the necessity to test experimentally the functionality of the predicted aptamers before annotating them solely based on sequence or secondary structure conservation.

Thiamine is scarce in oceanic surface waters (Sañudo‐Wilhelmy *et al*., 2012) and it is thought of as being growth limiting for some primary producers in certain environments (Paerl *et al*., 2015), with special relevance for harmful algal species (Tang *et al*., 2010). In this context, the experimental confirmation of an HMP‐P synthase and a thiamine transporter conserved in most of the available diatom genomes is of significant ecological relevance, given that these algae are responsible for 20% of global primary production (Field *et al*., 1998; Rousseaux & Gregg, 2014). The ubiquitous presence of genes encoding thiamine transporters and for the full thiamine biosynthesis pathway in the analysed diatom genomes did not offer sufficient information to hypothesise whether and under which conditions diatoms are net suppliers or consumers of thiamine and/or its moieties. The supply of thiamine and its moieties in oceanic environments has been shown to be dynamic and complex (Carini *et al*., 2014), and further research is needed to understand the ecological flows of this critical micronutrient in oceanic communities. Additionally, we have provided evidence to propose that genes encoding NMT1 domains found in several diatom, cryptophyte and haptophyte genomes are potentially involved in pyrimidine salvage. This is of special relevance given that some algal species have been shown to be dependent on only one of the thiamine moieties (Gutowska *et al*., 2017), and that some marine bacterial species can grow on HMP but not on thiamine (Carini *et al*., 2014). Finally, we have found predicted TPP aptamers associated with most *THIC* and *SSSP* genes. Although our results showed they are not responsive to thiamine supplementation under our laboratory conditions in *P. tricornutum* and *T. pseudonana*, we cannot rule out that they have a conserved function significant for the regulation of thiamine metabolism with implications for thiamine dynamics in oceanic communities.

In summary, the findings presented here expand our knowledge on how thiamine is produced and taken up by diatoms and show that the regulation of thiamine metabolism is more complex than previously thought. Further research will allow us to understand the full ecological and environmental implications of these findings in diatoms, a key taxonomic group in marine ecosystems and the main oceanic primary producers.

## Competing interests

None declared.

## Author contributions

ML‐P and AGS conceived and designed the research; ML‐P, KG, AH, PM and AGS planned the experimental work; ML‐P, KG, PM, AH, SAN, MPD and GIM‐O performed the experiments and data analysis; ML‐P and AGS wrote the manuscript with contributions from all authors. All authors reviewed and accepted the submitted manuscript.

## Supporting information


**Fig. S1** Phylogenetic tree and multiple sequence alignment for algal gene candidates with NMT1 domains.
**Fig. S2** Effect of thiamine and 4‐amino‐5‐hydroxymethyl‐2‐methylpyrimidine on NMT1 domain‐containing gene transcript levels in *Phaeodactylum tricornutum* and *Thalassiosira pseudonana*.
**Fig. S3** *Chlamydomonas reinhardtii* and *Phaeodactylum tricornutum* intracellular thiamine and thiamine pyrophosphate levels under increasing extracellular thiamine concentrations.
**Fig. S4** 3′RACE RT‐PCR on *PtTHIC* in the presence or absence of 10 μM thiamine or 4‐amino‐5‐hydroxymethyl‐2‐methylpyrimidine.
**Fig. S5** Characterisation of NMT1 domain‐containing gene knock‐out mutants generated by CRISPR/Cas9.

**Table S1** Thiamine pyrophosphate riboswitch prediction in diatom genomes.

**Table S2** Diatom genomes analysed in this study.

**Table S3** Identification of thiamine‐related genes in diatom genomes.

**Table S4** Primers used in this study.
Please note: Wiley Blackwell are not responsible for the content or functionality of any Supporting Information supplied by the authors. Any queries (other than missing material) should be directed to the *New Phytologist* Central Office.

## Data Availability

All raw data, query sequences and scripts to generate the figures in this paper can be found in the GitHub online repository: https://github.com/AndreHolzer/Llavero‐Pasquina_et_al_2022.
